# Palliative treatment of bone metastases: analysis of biological effects of MR guided Focused Ultrasound (MRgFUS) versus External Beam Radiation Therapy (EBRT). A randomized comparative trial using Functional Diffusion Maps as molecular activity indicator

**DOI:** 10.1186/2050-5736-2-S1-A17

**Published:** 2014-12-10

**Authors:** Michele Anzidei, Alessandro Napoli, Giulia Brachetti, Maurizio Del Monte, Daniel De Olivera, Fabrizio Andrani, Carola Palla, Luca Bertaccini, Daniela Musio, Vincenzo Tombolini, Carlo Catalano

**Affiliations:** 1 Department of Radiological Oncological and Pathological Sciences, “Sapienza”Unversity of Rome, Italy

## Backgroud

New functional and metabolic imaging techniques may provide the ability to assess biological changes that act as indicators of therapy response [1,2]. Particularly, Diffusion-Weighted Imaging (DWI) can be used to monitor change in cellularity within the tumour over time, which is reflective of response to therapy [3-5]. Our study aims to investigate and compare the response to MR guided Focused Ultrasound (MRgFUS) and External Beam Radiation Therapy (EBRT) treatments of painful bone metastases by using DW Magnetic Resonance imaging (DW-MRI) with apparent diffusion coefficient (ADC).Furthermore, we analyzed the correlation between DW-MRI and the Visual Analogue Scale (VAS).

## Materials and methods

This prospective, double arm, randomized study with EBRT serving as control arm, received IRB approval. 36 consecutive patients (female:15 male:21 mean age:60.3) with painful bone metastases were enrolled. 18 patients underwent MRgFUS treatment, using ExAblate 2100 system (InSightec), and 18 patients underwent EBRT. Pain palliation was evaluated by visual analog scale (VAS), pain questionnaires and changes in the patients’ medication. All patients underwent 3T ce-MRI (Discovery 750, GE; gd-BOPTA, Bracco) before treatment and at 1, 2, and 3 months afterward. MRI protocol included DWI sequences with five *b* factors (0–800 s/mm^2^) and ADC were obtained. The average ADC values for each lesion were analyzed in comparison between pre- and post-treatment.

## Results

No treatment-related adverse events were recorded for both arms. Statistically significant difference between baseline and follow-up VAS values and medication intake for both MRgFUS and EBRT patients (p<0.05) was noted. DWI showed substantial increase in mean ADC values after treatment for both MRgFUS (pre:1080,6±269 mm²/s; post: 1577,5±311,6) and EBRT (pre:1313,3±424,3 mm²/s; post:1777,9±386,3); there were no significant statistical differences in ADC shift following between MRgFUS and ERBT (p>0.5). Progressive decrease in VAS values positively correlated to an increase in mean ADC values (p>0.01) for both treatment modalities (Tables 1, 2).

**Table 1:** 

MRgFUS	baseline	Follow-up 1 month	Follow-up 2 months	Follow-up 3 months
**ADC**	1080.6±269 mm^2^/s	1347.2±294 mm^2^/s	1428.7±306.7 mm^2^/s	1577.5±311.6 mm^2^/s

**VAS**	7.09±1.8 (range 4-10)	2.65±1.36 (range 0-5)	1.04±1.91 (range 0-6)	1.09±1.99 (range 0-6)

**Table 2:** 

EBRT	baseline	Follow-up 1 month	Follow-up 2 months	Follow-up 3 months
**ADC**	1313.3±424.3mm^2^/s	1463±361.7 mm^2^/s	1611±373.2 mm^2^/s	1777.9±386.3 mm^2^/s

**VAS**	6.12±1.1 (range 5-10)	3.51±1.54 (range 0-6)	1.32±1.48 (range 0-4)	1.02±1.36 (range 0-4)

## Conclusions

**Figure 1:**
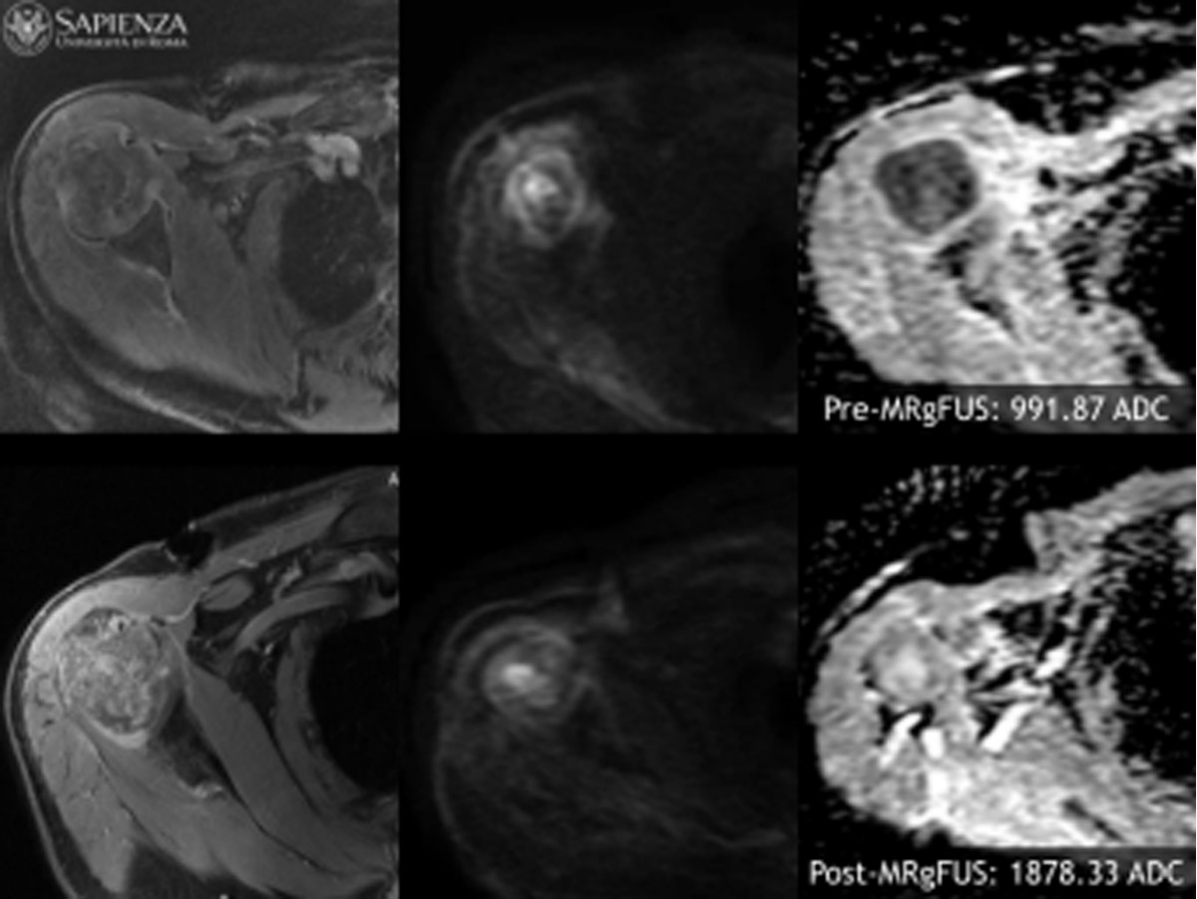
67 years old patient with lung cancer. MR images acquired before and after MR guided ultrasound treatment shows increase ADC value at 3 months follow-up (1878.33 mm^2^/s) compared to baseline (991.87 mm^2^/s). Also at the end of follow-up we observed absence of pain (VAS baseline: 7; VAS 3 months: 0).

**Figure 2:**
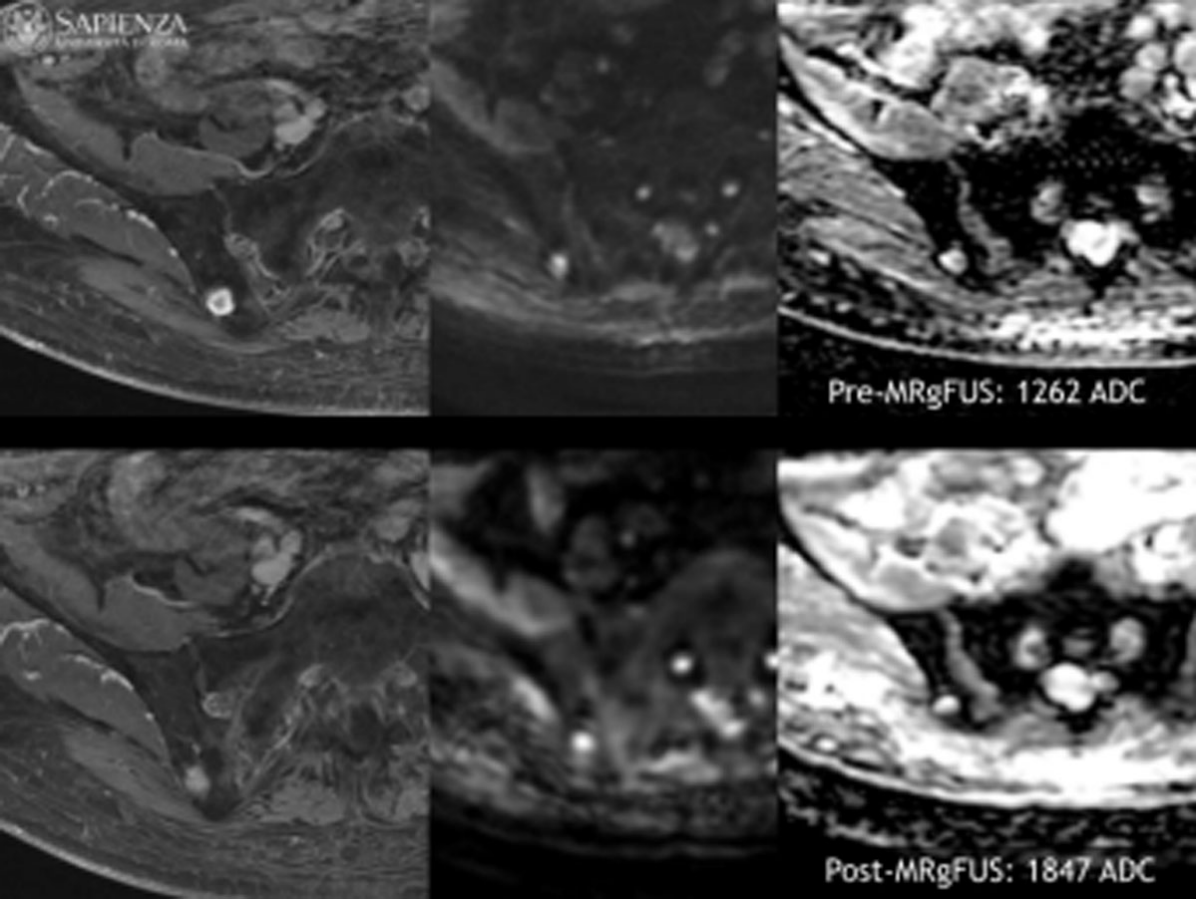
70 years old patient with renal cell carcinoma. Same as figure 1, it has been verified an increase ADC value after MR guided ultrasound treatment (ADC baseline: 1262 mm^2^/s. ADC 3 months: 1847 mm^2^/s). At 3 months follow-up VAS value 4 (VAS baseline 8).

DW-MRI with ADC is a viable option for assessing tumor response to MRgFUS therapy for bone metastases. The increase in mean ADC values of ablated bone metastases could reflect pathophysiologic alterations that occur after ablative therapy [6]. It is therefore possible that ADC variations could effectively describe and express effects on tumor mass control induced by focused ultrasound ablation. MRgFUS is a promising noninvasive treatment modality for successful palliation of bone metastasis [7] and has potential for tumor control (Figures 1, 2) [8] as compared to ERBT. MRgFUS determines bone metastasis cell damage similarly to EBRT as demonstrated by linear ADC modification.
